# Feeding Strategies to Mitigate Enteric Methane Emission from Ruminants in Grassland Systems

**DOI:** 10.3390/ani12091132

**Published:** 2022-04-28

**Authors:** Juan Vargas, Emilio Ungerfeld, Camila Muñoz, Nicolas DiLorenzo

**Affiliations:** 1 Department of Animal Sciences, North Florida Research and Education Center, University of Florida, Marianna, FL 32443, USA; jvargasmartinez@ufl.edu; 2 Centro Regional de Investigación Carillanca, Instituto de Investigaciones Agropecuarias, Temuco 4880000, Chile; emilio.ungerfeld@inia.cl; 3 Centro Regional de Investigación Remehue, Instituto de Investigaciones Agropecuarias, Osorno 5290000, Chile; camila.munoz@inia.cl

**Keywords:** diet supplementation, grassland systems, grazing management, methane emission, secondary compounds tannin-containing legume

## Abstract

**Simple Summary:**

Ruminants under grazing conditions play an important role, especially in developing countries. Enteric methane emissions from ruminants are greater with pasture-based diets; however, it is not clear which abatement practices are effective to reduce methane emissions under grazing conditions. The objective of this review was to identify and describe enteric methane abatement practices for ruminants that are applicable under grazing conditions. Decreasing the pre-grazing herbage mass reduced methane emissions per unit of product. Other grazing management practices such as increased stocking rate, decreased forage maturity, rotational stocking, and incorporating tannin-containing or non-tannin-containing legumes showed inconsistent results. Nitrogen fertilization or silvopastoral systems did not modify methane emissions, although they may alter carbon sequestration in a system. Supplementation in grazing conditions shows inconsistent responses on methane emissions. However, lipid supplementation showed promising results. Identifying and implementing grazing strategies and supplementation practices under grazing conditions is required to increase efficiency and reduce the environmental impact of these systems.

**Abstract:**

Ruminants produce approximately 30% of total anthropogenic methane emissions globally. The objective of this manuscript was to review nutritional enteric methane abatement practices for ruminants that are applicable under grazing conditions. A total of 1548 peer-reviewed research articles related to the abatement of enteric methane emissions were retrieved and classified into four categories: non-experimental, in vitro, in vivo confined, and in vivo grazing. The methane abatement strategies for grazing systems were arranged into grazing management and supplementation practices. Only 9% of the retrieved papers have been conducted under grazing conditions. Eight grazing management practices have been evaluated to reduce methane emissions. Decreasing the pre-grazing herbage mass reduced the methane emission per unit of product. Other grazing management practices such as increased stocking rate, decreased forage maturity, rotational stocking, and incorporating tannin-containing or non-tannin-containing feeds showed contradictory results. Nitrogen fertilization or silvopastoral systems did not modify methane emissions. Conversely, supplementation practices in grazing conditions showed contradictory responses on methane emissions. Lipid supplementation showed promising results and suggests applicability under grazing conditions. Identifying and implementing grazing strategies and supplementation practices under grazing conditions is required to increase efficiency and reduce the environmental impact of these systems.

## 1. Introduction

Ruminants are an essential component of grassland ecosystems. They maintain the dynamic cycle of nutrients through grazing and nutrient excretion [1]. Additionally, they convert non-edible feeds into high-quality food for human nutrition [2], promote human food security, especially in developing communities, and play an essential role in cultural and social relationships in many societies [3]. Grasslands support 360 million cattle and more than 600 million sheep and goats worldwide [4]. However, ruminant production in grassland ecosystems has been associated with deforestation, biodiversity loss, and water and air contamination [5,6].

Globally, livestock produce 14.5% of total anthropogenic emissions of greenhouse gases (GHG), with enteric methane (CH_4_) being the primary source (39.1% [7]). Further, it has been proposed that 47% of CH_4_ emissions are the result of ruminants under grazing conditions [8], associated with poor animal performance [9]. For this reason, these systems produce more CH_4_ per unit of product than mixed or confined production systems [10,11]. Regardless of the production system, CH_4_ emissions represent an energy loss for ruminants, varying between 2 and 12% of gross energy intake [12].

Enteric CH_4_ can be expressed in different forms: as an absolute amount emitted per day (i.e., total CH_4_ production), relative to the unit of dry or organic matter consumed (i.e., CH_4_ yield), or by product produced such as meat or milk (i.e., CH_4_ intensity), either as a percentage of the gross energy ingested (i.e., CH_4_ conversion factor, Ym) or relative to the unit of grazed area.

Strategies for decreasing CH_4_ emissions from ruminant livestock can be grouped into animal management, genetic selection, rumen microbiome manipulation, and nutritional modulation [11,13,14]. Practices that promote more efficient systems due to better animal performance or fewer inputs reduce CH_4_ intensity [2,10,15]. In the past decades, various strategies have been evaluated both under in vitro and in vivo conditions. In vitro techniques are convenient for evaluating many samples and understanding the chemical and biological mechanisms involved in CH_4_ production [16]. In vivo methodologies allow a more holistic approach and can take place in confined or grazing conditions. Confined methods allow better control of experimental procedures, particularly feed intake, and often provide more precise results [17]. However, research under confined conditions has limitations when extrapolated to grazing conditions because it does not convey all the complexity and dynamic interactions that occur between soil, forage, animal, herd, and climate [18]. In addition, confined experimental conditions to measure enteric methane (e.g., chambers) could decrease dry matter intake by ruminants, potentially affecting the CH_4_ emission determination [19,20].

Animal response in grazing systems is affected by environmental, management, social, and individual factors that differ from confined systems [21]. Environmental conditions such as heat can modify ruminant grazing patterns [22,23]. Further, grazing management modulates animal forage selection and nutrient recycling, ultimately impacting herbage mass accumulation and forage nutritive value [1]. Additionally, herd interaction alters consumption behavior associated with animal hierarchy, and ruminants show an individual grazing behavior associated with previous experience or modulated through epigenetic stimuli [21]. Because intake is the main driver of CH_4_ production [12,17], the differences between grazing and confinement may be partially explained by variations in dry matter intake. However, the type of ruminal fermentation in both systems (i.e., proportion of propionate relative to other VFA) may have a greater impact on the differences in methanogenesis, assuming the predominance of cereal-based diets in confinement.

Determining enteric CH_4_ emissions in grasslands poses significant challenges as few in vivo techniques are available that can accurately measure daily CH_4_ emissions under grazing conditions [18,24]. Constraints in measuring variables related to CH_4_ emissions (e.g., dry matter intake) and handling ruminants with a specific frequency, especially in extensive systems, limit animal interventions such as the use of feed additives or supplementation [14,25,26]. This manuscript aims to critically review nutritional abatement strategies of enteric CH_4_ from ruminants in grassland systems reported in the scientific literature.

## 2. Research on Methane Emission Abatement Strategies

Peer-reviewed publications were obtained from ScienceDirect (https://www.sciencedirect.com/ accessed on 11 October 2021), Springer (https://www.springer.com/us accessed on 18 October 2021), and Scielo (https://www.scielo.org/ accessed on 5 October 2021), using the following keywords: “methane”, “methane emissions”, “methane production”, and “ruminant”. A total of 1548 documents from 1956 to 2020 were retrieved and classified into four categories as follows:

i. Non-experimental: Represented by documents that did not involve original research data collection, for example, reviews, meta-analyses, life cycle assessments, inventory estimations, or methodology description.
ii. In vitro: This category comprised research that evaluated CH_4_ emissions in batch or semi-continuous in vitro cultures.
iii. Confined studies: Represented by documents that evaluated in vivo CH_4_ emissions, where ruminants were restricted to confined facilities.
iv. Grazing studies: This category includes research that determined in vivo CH_4_ emissions under grazing conditions.

Retrieved documents were categorized according to the continent where the experiments were conducted (i.e., the Americas, Africa, Asia, Europe, or Oceania), production system (i.e., beef, dairy, or small ruminants), type of forage (i.e., temperate or tropical forage), and CH_4_ measurement technique.

**Figure 1:**
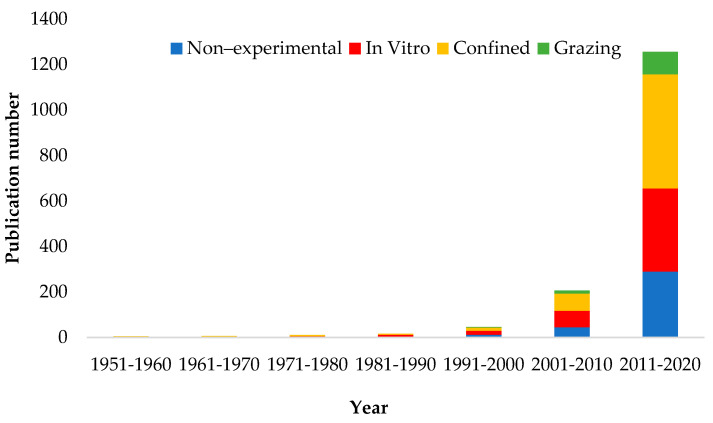
Publishing decade of retrieved peer-review documents of enteric methane emissions from ruminants.

Research evaluating CH_4_ emissions from ruminants published in the last decade increased by 6 and 28-fold relative to the decades 2001–2010 and 1990–2000, respectively (Figure 1). Thus, there has been an increase in interest in measuring and evaluating strategies to reduce CH_4_ emissions from ruminants in the last decade. Beauchemin et al. [25] reported similar tendencies, emphasizing that early CH_4_ research focused more on increasing animal energy efficiency, understanding methanogenesis biochemical pathways, and evaluating rumen modifiers under in vitro or confined conditions. After the development of the sulfur hexafluoride (SF_6_) tracer technique [12], and more recently, the use of the GreenFeed (GF, C-Lock Inc., Rapid City, SD) technology [27], more experiments have been conducted under grazing conditions [25].

Publications involving reviews, meta-analyses, methodology description, and theoretical analyses (i.e., non-experimental category) represented 23% of the retrieved documents. In vitro publications were 31% of the documents, with the batch culture methodology being predominant (reported in 83% of the in vitro studies). The in vivo Confined category represented 39% of the documents, and respiration chambers, SF_6_, and GF techniques used in confinement studies were 57, 20, and 7% of the documents, respectively. Further, 16% of the confined studies provided cut and carry forage as a proxy for grazing conditions.

**Figure 2:**
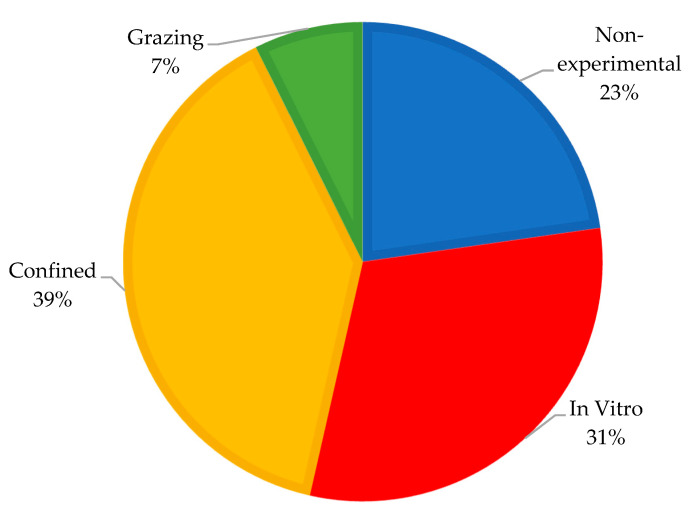
Distribution (%) of retrieved peer-review documents into Non-experimental, In vitro, Confined, and Grazing categories of methane emissions from ruminants.

**Figure 3:**
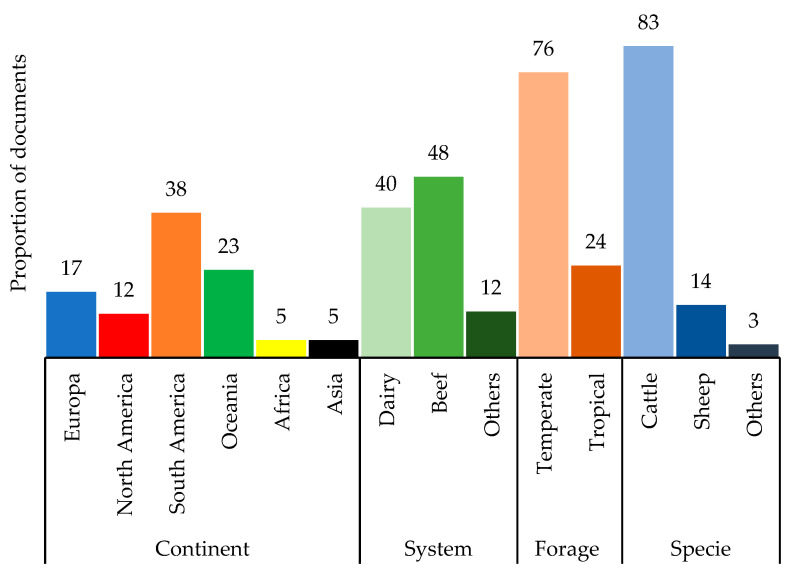
Distribution (%) of publications from the grazing category classified by continent, production system, forage type, and ruminant species.

The in vivo Grazing category represented 7% of the documents (Figure 2), and the SF_6_ technique was used in 70% of the grazing research on CH_4_ abatement. Research on enteric CH_4_ emissions under grazing conditions has been carried out mainly in Europe and the Americas (67%), evaluating mostly cattle (83%) under temperate pastures (76%) (Figure 3).

## 3. Methane Emissions in Grassland Systems

Grassland systems vary from extensive, generally with low productivity per area, low forage nutritive value, non-improved ruminant breeds, and without supplementation schemes, to intensive, generally with high productivity, improved animal breeds, grasses, and management, and more balanced diets [7,26]. This diversity in production systems requires the development of different approaches to reduce enteric CH_4_ emissions that will have a minimal effect on farm labor activities, production costs, and profitability. For example, continuous inclusion of a feed additive may be easily incorporated in intensive grazing systems through supplemental feed (e.g., during the milking routine). However, in extensive grazing conditions, daily additive supplementation is less feasible, and other strategies must be developed to ensure continuous CH_4_ abatement.

From a feed and nutritional perspective, strategies to decrease CH_4_ emissions can be classified into practices related to grazing management or strategic supplementation of ingredients or additives. Managing grazing intensity—targeted through contrasting stocking rates or pre-grazing herbage masses—and concentrate supplementation have been the most reported strategies to modulate CH_4_ under grazing conditions (12 and 9 studies, respectively). This was followed by lipid (6 studies) and nitrate supplementation (3 studies). Other strategies that modify forage nutritive value (i.e., incorporation of tannin-containing legumes or manipulation of forage maturity) have been less evaluated (2 studies each).

### 3.1. Grazing Management Strategies to Mitigate Methane Emissions from Ruminants

Grazing management consists of practices that manipulate forage characteristics to pursue one or multiple objectives. Grazing intensity, phenological stage of grasses, and grazing method are management tools that manipulate forage availability or quality [28]. Grazing intensity, generally expressed as stocking rate, or relationship between the number of animals and amount of land grazed, is the main factor affecting nutrient cycling, and defines animal productivity while delivering ecosystem services [28,29]. Under grazing conditions, increasing the stocking rate showed inconsistent results on CH_4_ emissions (Table 1). However, methane intensity was not affected by increasing the stocking rate (Table 1).

Increasing the stocking rate decreases forage biomass, but increases forage nutritive value [59], as greater stocking rates are associated with extensive biomass defoliation, maintaining the vegetative stage of forages [28]. Consumption of forage material in a vegetative stage is associated with reduced CH_4_ yield due to a lesser concentration of cell wall structure [43]. Additionally, intake of forages in a vegetative stage increases dry matter consumption, decreases ruminal retention time, and reduces CH_4_ yield (i.e., grams of CH_4_ per unit of dry matter intake), although it could increase total CH_4_ production (i.e., total grams of CH_4_ per animal per day [60]). Conversely, individual animal productivity decreases when the stocking rate increases due to greater grazing competition and less possibility for animal forage selection [61]. The intensity or yield of enteric CH_4_ emissions may be increased with high stocking rates if forage availability is restricted and is insufficient to meet the animal’s nutrient requirements.

The phenological stage of grazing is related to the physiological stage of forage, i.e., forage maturity, when the defoliation occurs [28]. Reduced forage maturity decreased the CH_4_ conversion factor (i.e., Ym; Table 1). Mature forages have a lesser concentration of digestible tissues such as parenchyma and more cell wall structure. This structure is mainly composed of cellulose, hemicellulose, and lignin and has slow rumen fermentation and passage rates [62]. The fermentation of cell wall carbohydrates yields more CH_4_ than non-structural carbohydrates [63]. Conversely, immature forages have more degradable nutrients and result in increased dry matter intake [42]. Thus, it is expected that mature forages in ruminant diets produce a greater CH_4_ yield due to greater cell wall concentration. However, if the forage cell wall structure limits intake (i.e., physical restriction), the CH_4_ production may be reduced, increasing CH_4_ yield [60].

The grazing method refers to how animals are stocked and is generally classified as continuous or rotational [64]. Usually, rotational grazing is associated with more uniformly distributed grazing and manure deposition, increasing the carrying capacity and efficiency of grazing, maintaining forage uniformity, conserving nutrient soil characteristics, and increasing productivity per unit of area [28,65]. In contrast, continuous grazing promotes greater herbage selection and intake if animals have a similar herbage allowance [31]. In our review, the grazing method did not show consistent results on CH_4_ emissions (Table 1). In one experiment, increased CH_4_ intensity was associated with decreased animal production in rotational vs. continuous stocking, related to lower intake [31]. Under well-managed grazing conditions, no differences were observed in continuous or rotational grazing in herbage accumulation, forage nutritive value, intake, or performance because the animal can select and consume forages of greater nutritional value [64]. The limited number of studies comparing continuous and rotational grazing limits the evaluation of the effects of rotational or continuous methods on CH_4_ emissions. Additionally, to our knowledge, no studies have evaluated other grazing methods such as first-last grazers [64] on CH_4_ emissions.

Another strategy to increase herbage mass is applying nitrogen fertilization [54,66], allowing a greater stocking rate and extending the grazing period [59]. Nitrogen fertilization did not modify enteric CH_4_ production except with nitrogen application rates greater than 400 kg/ha [45]. However, nitrogen application did not modify forage biomass or nutritive value, explaining the absence of effects on CH_4_ yield or intensity (Table 1). No experiments have evaluated the effects of fertilization with other nutrients on CH_4_ production.

Legume inclusion in pastures has important advantages for grassland systems. Legumes can fix atmospheric nitrogen through the symbiotic relationship with *Rhizobium* bacteria and increase crude protein concentration in forage diets [67]. Legumes are C3 species and have more digestible tissue (i.e., mesophyll), greater crude protein concentration, and greater microbial degradation than C4 grasses [62,68]. Several studies have been conducted to evaluate the effects of legumes on CH_4_ production. There are differences in ruminant CH_4_ emissions when the legume evaluated contains condensed tannins. Generally, non-tanninferous legumes such as *Medicago sativa* or *Trifolium* spp. Have a greater extent of ruminal organic matter digestion, especially in low-quality grass-based diets [50]. Their inclusion in ruminants’ diets increase dry matter intake and animal performance [48,52,53,69]. However, in most retrieved manuscripts, non-tanninferous legumes did not modify CH_4_ production, yield, or intensity (Table 1). Factors such as legume proportion in pasture, dry matter intake, organic matter digestibility, and passage rate can help explain the absence of any effects on CH_4_ emissions. For example, McCaughey et al. [52] reported that 78% of alfalfa in the pasture increased dry matter intake by 13% and decreased CH_4_ production by 10%. In contrast, Chaves et al. [49] reported that 40% of alfalfa in the pasture did not affect intake or CH_4_ production.

Tannin-containing legumes such as *Lotus* spp. or *Calliandra calothyrsus* contain polyphenol compounds that protect plants against external stressors. Variations in tannin type, concentration, and activity depend on environmental conditions and management practices [70]. Waghorn [71] suggested that tannins reduce ruminal protein and carbohydrate fermentation and microbial enzyme activity and affect methanogenic archaea populations [70,72]. Thus, CH_4_ emissions may be reduced due to decreased nutrient fermentation, diminished dihydrogen (H_2_) production, or a modified archaeal community. However, high tannin concentration is associated with detrimental effects on animal performance, related to decreased dry matter intake and protein digestibility [71]. There is too little information to conclude on the effect of the inclusion of tannin-containing legumes as a strategy to modify CH_4_ emissions (Table 1).

Silvopastoral systems are a spatial arrangement where multiple forage strata grow together to provide forage biomass and other ecosystem services [73]. There are multiple silvopastoral designs where woody species may or may not provide forage for ruminant diets [74]. Silvopastoral systems decrease the nutritive value of grass and biomass production because trees can intercept light, increase cell wall concentration, and reduce herbage photosynthetic ability [75]. However, in warm or dry conditions, the presence of trees produces a micro-environment that maintains forage production, reduces maturity, and increases nutritive value [73]. There are few evaluations of silvopastoral systems on enteric CH_4_ emissions. Ruminants in silvopastoral systems produced similar CH_4_ emissions to ruminants in grasslands without trees due to similar forage nutritive value and animal intake (Table 1). The foliage of some shrubs and trees has secondary compounds, such as tannins or saponins, that have been reported to decrease CH_4_ emissions from ruminants; however, this has not been evaluated under grazing conditions [76].

### 3.2. Supplementation Strategies to Mitigate Methane Emissions from Ruminants in Grasslands Systems

Diet supplementation is a nutritional strategy to supply deficient nutrients, improve the health status, increase animal performance, and reduce GHG emission intensity, especially in undernourished ruminants [77]. Concentrate supplementation at pasture showed contradictory results on CH_4_ emissions (Table 2). It is expected that concentrate supplementation increases rumen fermentation of forage diets, resulting in greater absolute production of H_2_ and CH_4_ in the rumen [78]. Thus, concentrate supplementation may increase the total CH_4_ production (i.e., total grams of CH_4_ per animal per day). Conversely, concentrate supplementation increases ruminal passage and reduces the rumen pH, resulting in lower CH_4_ relative to organic matter fermented because the hydrogen is redirected to other metabolic pathways (i.e., propionate or microbial bacteria synthesis; [79,80]. In addition, methanogens are sensitive to ruminal pH lower than 6 [81]. Thus, concentrate supplementation might decrease CH4 yield (i.e., total grams of CH_4_ per unit of OM fermented). Finally, concentrate supplementation increases dry matter intake and animal performance resulting in lower CH_4_ intensity (i.e., total grams of CH_4_ per unit of product), and effects appear to be dependent on the level of concentrate supplied.

Lipid supplementation is another strategy to increase the energetic density of ruminant diets, especially during high-energy demand periods such as early lactation [97], which has shown promise as a CH_4_ mitigation strategy [98]. Lipid supplementation under grazing conditions decreased CH_4_ intensity 60% (Table 2). Lipid inclusion may reduce fiber digestion, by coating the fiber against microbial fermentation [14,99]. Fiber fermentation is related to acetate and H_2_ production in the rumen. In this regard, reducing dry matter intake and fiber degradability potentially decreases H_2_ and CH_4_ production, though it is not a desirable mechanism to reduce CH_4_ emissions [78]. Furthermore, fatty acids can inhibit methanogens. Poly-unsaturated and medium chain fatty acids have toxic and disruptive effects on methanogen cell membranes [98,100]. In addition, unsaturated fatty acids capture H_2_ during the rumen biohydrogenation process, although this represents a small proportion of H_2_ capture [12]. Finally, nitrates are reduced in the rumen to ammonia, competing with methanogenesis [9]. However, nitrates did not reduce CH_4_ emissions of ruminants under grazing conditions (Table 2).

## 4. Perspectives on Methane Mitigation Strategies in Grasslands Systems

Designing effective mitigation strategies of CH_4_ emissions under grazing conditions represents a significant challenge, especially in extensive systems. These challenges are reflected in the fact that considerably less research has been conducted under grazing conditions, in which there is less control of the experimental conditions compared to confined systems. In addition, the heterogeneity and seasonal variation in grazing lands’ forage composition and growth further complicate conditions in these systems by adding experimental variation. The most widespread technique for measuring CH_4_ under grazing conditions is the SF_6_ tracer technique, which is less precise and more labor intensive than respiration chambers [17,18]. The inability to accurately determine intake in grazing conditions hinders the ability of researchers in terms of assessing CH_4_ emissions yield and the impact of interventions on forage intake. Thus, results of CH_4_ abatement strategies evaluated in grazing systems are less consistent than in confined studies, and researchers may need to rely on the impact on emissions intensity rather than yield when assessing these strategies. Animal performance (i.e., milk yield or growth) is frequently and more precisely evaluated during grazing experiments. Therefore, CH_4_ intensity should be a more useful variable to compare practices in grazing conditions.

This review highlights CH_4_ mitigation practices that have been studied under grazing conditions. Emission of CH_4_ may be reduced through grazing practices that modify the forage composition (i.e., reducing structural carbohydrate intakes) as a result of an increased stocking rate or lower pre-grazing herbage mass. Rotational grazing does not increase emissions intensity unless animal intake is restricted, compromising production. Decreasing forage maturity and the presence of tannin-containing legumes can decrease CH_4_ emissions; however, more research is required under grazing conditions to further quantify this. Non-tanniferous legumes mostly did not modify CH_4_ emissions. Additionally, nitrogen fertilization and silvopastoral systems have had no effects on CH_4_ emissions. Conversely, concentrate and lipid supplementation of grazing diets have improved animal performance and reduced CH_4_ intensity. Nitrates supplementation has not shown a consistent effect on CH_4_ production from grazing ruminants. Supplementation can be problematic in extensive systems where infrequent animal management and low profitability restricts its implementation [11,25]. Other additives such as tannins, 3-nitrooxypropanol, or red algae have shown promising results on CH_4_ reduction under confined conditions [101,102], but their effects on grazing systems are as yet uncertain. Finally, long-term studies and integrative evaluation through life cycle assessment analysis are needed to generate technologies that promote greater biological efficiency and farm profitability while reducing the detrimental effects on the environment.

## Figures and Tables

**Table 1:** Number of experiments that reported either increased, decreased, or not modified CH_4_ emissions when implementing different grazing management practices.

Grazing or Pasture Management	CH_4_ g/d		CH_4_ g/kg DM or OM		CH_4_ g/kg Product		CH_4_ %GEI ^1^		Source
	Effect	NE ^2^	Effect	NE ^2^	Effect	NE ^2^	Effect	NE ^2^	
Increasing Stocking Rate	Increase	0	Increase	0	Increase	0	Increase	0	[30,31,32,33,34,35]
	No effect	5	No effect	4	No effect	3	No effect	6	
	Decrease	5	Decrease	3	Decrease	0	Decrease	1	
Decreasing pre-grazing herbage mass	Increase	2	Increase	1	Increase	1	Increase	1	[36,37,38,39,40,41]
	No effect	4	No effect	3	No effect	2	No effect	2	
	Decrease	2	Decrease	3	Decrease	5	Decrease	3	
Decreased forage maturity	Increase	0	Increase =	0	NR ^3^	NR ^3^	Increase	0	[42,43]
	No effect	1	No effect	1			No effect	0	
	Decrease	1	Decrease	1			Decrease	2	
Rotational systems	Increase	0	Increase	0	Increase	2	Increase	0	[31,34,44]
	No effect	5	No effect	1	No effect	1	No effect	2	
	Decrease	2	Decrease	1	Decrease	0	Decrease	1	
N fertilization	Increase	0	Increase	0	Increase	0	Increase	0	[45,46,47]
	No effect	3	No effect	1	No effect	2	No effect	1	
	Decrease	1	Decrease	0	Decrease	0	Decrease	0	
Inclusion of non-tannin-containing legumes into the pastures	Increase	1	Increase	1	Increase	0	Increase	1	[46,48,49,50,51,52,53]
	No effect	4	No effect	4	No effect	4	No effect	2	
	Decrease	1	Decrease	1	Decrease	0	Decrease	1	
Inclusion of tannin-containing legumes into the pastures	Increase	1	Increase	0	Increase	0	Increase	0	[54,55]
	No effect	1	No effect	1	No effect	1	No effect	0	
	Decrease	0	Decrease	1	Decrease	1	Decrease	1	
Silvopastoral systems	Increase	0	Increase	0	Increase	0	Increase	0	[47,56,57,58]
	No effect	6	No effect	2	No effect	2	No effect	2	
	Decrease	0	Decrease	0	Decrease	0	Decrease	0	

^1^. GEI = Gross energy intake; ^2^. NE = Number of experiments; ^3^. NR = Not reported.

**Table 2:** Number of experiments that reported either increased, decreased, or not modified CH_4_ emissions when supplementing concentrates or nitrates to ruminants under grazing conditions.

Supplementation Strategy	CH_4_ g/d		CH_4_ g/kg DM or OM		CH_4_ g/kg Product		CH_4_ %GEI ^1^		Source
	Effect	NE ^2^	Effect	NE ^2^	Effect	NE ^2^	Effect	NE ^2^	
Concentrate inclusion	Increase	5	Increase	0	Increase	0	Increase	0	[37,42,44,82,83,84,85,86,87,88,89]
	Equal	8	Equal	9	Equal	7	Equal	8	
	Decrease	2	Decrease	3	Decrease	2	Decrease	3	
Lipid supplementation	Increase	0	Increase	0	Increase	0	Increase	0	[90,91,92,93]
	Equal	4	Equal	3	Equal	2	Equal	3	
	Decrease	5	Decrease	4	Decrease	3	Decrease	2	
Nitrate supplementation	Increase	0	Increase	0	Increase	0	Increase	0	[94,95,96]
	Equal	3	Equal	3	Equal	2	Equal	2	
	Decrease	0	Decrease	0	Decrease	0	Decrease	0	

^1^. GEI = Gross energy intake; ^2^. NE = Number of experiments.

## Data Availability

The data presented in this paper are available on request from corresponding author.
